# The Voluntary Hospitals Made Secure

**Published:** 1920-02-07

**Authors:** 


### The Hospital

The Workers' Newspaper of Administrative Medicine and Institutional Life, Administration, National Insurance and Health.

No. 1757, Vol. LXVII. SATURDAY, FEBRUARY 7, 1920. PRICE TWOPENCE

### THE WAY TO WIN HOME.

### The Voluntary Hospitals Made Secure.

When we began our crusade of courage and hope, as an inspiration to all Voluntary Hospital managers throughout the country, and to those who had the sound judgment to make themselves familiar with the facts, and to realise that the Voluntary System is the one hospital system in the world which is undoubtedly the best, there were many sceptics and doubting hearts who, according to their habit of mind, began to use their energies in applying a cold douche to the more active of their fellows, who had faith and were prepared to work. Our crusade has been blessed in a wonderful way, and already, within three months, the whole of the hospital world in Great Britain, and especially in England and Wales, has sprung into activity. Wherever the managers have set to work quickly and with a will, success has come with a speed and completeness, that proves the Voluntary System rests secure in the hearts of the people of this country, as it undoubtedly should. Week by week new schemes have been evolved; more and more institutions have got to work; and this week we have a new and remarkable development, from that centre of active business progress and yield, Leeds, whose inhabitants have the grit, the will, and the force to achieve results, whenever they have put their hearts into the effort.

Mr. Braime, a member of the Board of the Leeds General Infirmary Special Finance Committee, has brought a scheme before the employers of labour for the raising of a settled amount of contributions to the hospital from each firm, employing both large and small numbers in their works and businesses. Mr. Braime set out to try and bring every firm in all trades into his scheme, so that each may contribute in equity, and bear its fair share in supporting these splendid institutions, our Voluntary Hospitals. These latter would thus be relieved of the harassing thought of where the next week's wages and expenses are to come from, and would possess, in the case of the Leeds General Infirmary and other important medical institutions in that city, a sure and reliable portion of their income, which can be depended upon, instead of relying on the spasmodic and uncertain gifts, donations, collections, etc., they have in the past had to anticipate. Mr. Braime has found that the employers, with wonderful unanimity, have approved his proposals, and that in many cases firms are willing to contribute 4s. per employee per annum, and in all other cases at least 2s. per employee per annum. His proposals have advanced far enough to enable it to be stated that his scheme is popular, and that under it, with the co-operation of the employers, there is now good reason to believe that the Leeds General Infirmary will soon be placed upon a sound financial basis, and be enabled to deal with a larger number of patients —approximately 600 to 1,000 now always waiting admission—many of whom have died in the past owing to the impossibility of treating them in the then existing conditions.

In directing our readers' attention to the article on pages 437-440 inclusive, where the Leeds plan is fully explained, and the results to date, actual and probable, are clearly set forth, we commend it to the attention of every hospital manager throughout Great Britain. We welcome and offer many grateful thanks to Mr. Braime and all Leeds people who have brought this new development to so successful a head. It once more demonstrates that, so far from the Voluntary Hospital Finances in Great Britain being in a parlous condition, wherever the management is in the hands of citizens who are business men with large hearts and good organising powers, a new and better system of finance for the Voluntary Hospitals is assured, and the £25,000,000, which we estimated as a sum likely to be required to finance the Voluntary Hospitals, is well in sight and can be readily obtained under proper organisation and system between now and June 30 next. Leeds, Nottingham, and Cardiff have now placed the monetary difficulties of the Voluntary Hospitals in a clear light, and have demonstrated by results that the sum required throughout Great Britain can be readily obtained, and the hospitals put upon a firm financial basis under new systems. Such systems must provide that the yield of revenue shall be capable of supplying all the money needed, and put an end once and for all to the miseries, injustices, and abuses, with the torment and anxieties for the sick hospital patients on waiting-lists, which have during the war, become a cruel accompaniment to the many trials, of various kinds, which all have had to face.

Leeds, of course, is a big industrial city, and there are many others included in the 250 centres with which we have been in correspondence, and have dealt in this series of articles. What Leeds has well in hand to accomplish, all these other centres can with equal readiness secure, providing they have the men, the brains, and the will to set to work and complete their organisation, with all possible despatch. But there are other schemes which have proved equally successful, as in the case of Nottingham, where an additional revenue of £20,000 a year is promised to the hospital—£10,000 in subscriptions from the working classes, and £10,000 more from the citizens. In the smaller centres and the non-industrial centres, wherever there are parishes and churches, the Hospital Sunday Fund in London, during the past few years has shown, that, the collections in every parish on one day in the year, carried out with the co-operation of residents, even in the poorer districts, will yield £25 to £50 year by year. Then there is the new ground to be cultivated and the new friends to be recruited and got interested in the Voluntary Hospitals, namely, twenty million non-givers and non-workers for hospitals, in the past, many of whom we know from our investigations are only too anxious to lend a hand, if invited to do so, and work with a purpose.

As a word of cheer, therefore, to all hospital workers throughout Great Britain with whom we have had the pleasure of communicating, we this week are in a position to state our conviction, about which there is not a shade of doubt, if the will and the workers are everywhere organised, that at the end of the Five Momentous Months **the Voluntary Hospitals will be made secure.** And as a final *au revoir* inspiration let us say, as we enter the train in search of rest, the **£25,000,000 or £30,000,000 needed may certainly be provided in fulfilment of the combined efforts of the Great Army** now assembling to give their voluntary services for the hospitals. The recruits in this Army, led and cheered by the ability, energy, and self-denial of everyone attached to the Voluntary Hospitals, have made up their minds to put their backs into the work and lead the people of all classes, everywhere, to victory for themselves, as well as for their neighbours, in the day of sickness or accident, whenever it may befall any of the citizens.

Let the doubters spend a little time in examining the figures. Take Leeds, which, Mr. Braime anticipates, can readily contribute an additional and new income for the infirmary of £25,000 a year, or the equivalent, at 4 per cent., of a capital sum of £625,000. Take Nottingham, with its additional and new income of £20,000 a year, which, capitalised on the same basis, represents £500,000. Take Cardiff, where £40,000 of the £150,000 the Lord Mayor is raising will constitute the new revenue in that city; this capitalised amounts to £1,000,000. Then take 200 of the 250 centres we are communicating with, which include practically all the big hospitals in Great Britain. Average them out at £10,000 of entirely new revenue. That represents collectively £2,000,000 of new revenue for the 200 voluntary hospitals concerned, or an equivalent in capital of £50,000,000 sterling. Is there a free man or woman who will study what is here written, and ever again stand shivering on the doorstep, when they think of the sick and suffering poor without hospital care, because there is no money to support the hospitals and the rates must gobble them up? The rate-support men had better go to school again to be taught arithmetic, and somewhere and somehow acquire a robuster faith in their fellows throughout the land. There are other schemes which the wise can readily evolve in the writer's absence and be ready to confront him with on his return to work.

